# Study of Postprandial Lipaemia in Type 2 Diabetes Mellitus: Exenatide versus Liraglutide

**DOI:** 10.1155/2014/304032

**Published:** 2014-08-04

**Authors:** Maria Voukali, Irene Kastrinelli, Sapfo Stragalinou, Dimitra Tasiopoulou, Pinelopi Paraskevopoulou, Nicholas Katsilambros, Alexandros Kokkinos, Nicholas Tentolouris, Ioannis Ioannidis

**Affiliations:** ^1^Diabetes and Obesity Center, Konstantopouleio Hospital, 3-5 Agias Olgas Street, Nea Ionia, 14233 Athens, Greece; ^2^Biochemistry Laboratory, Konstantopouleio Hospital, 3-5 Agias Olgas Street, Nea Ionia, 14233 Athens, Greece; ^3^1st Department of Propaedeutic and Internal Medicine, Laiko General Hospital, Athens University Medical School, 17 Agiou Thoma Street, 115 27 Athens, Greece

## Abstract

Therapeutic approaches based on the actions of the incretin hormone GLP-1 have been widely established in the management of T2DM. Nevertheless, much less research has been aimed at elucidating the role of GLP-1 in lipid metabolism and in particular postprandial dyslipidemia. Exenatide and liraglutide are two GLP-1 receptor agonists which are currently available as subcutaneously administered treatment for T2DM but their chronic effects on postprandial lipaemia have not been well investigated. The aim of this study is to examine the effect of treatment with either liraglutide or exenatide for two weeks on postprandial lipaemia in obese subjects with T2DM. This study was a single-center, two-armed, randomized, controlled 2-week prospective intervention trial in 20 subjects with T2DM. Patients were randomized to receive either liraglutide or exenatide treatment and underwent a standardized meal tolerance test early in the morning after 10 h fast at baseline (visit 1, beginning of treatment) and after a two-week treatment period (visit 2). Exenatide and liraglutide both appear to be equally effective in lowering postprandial lipaemia after the first administration and after a two-week treatment. The mechanisms which lead to this phenomenon, which seem to be independent of gastric emptying, are yet to be studied.

## 1. Introduction

The growing incidence of type 2 diabetes mellitus (T2DM) is a major problem in the modern world [1]. Most individuals with T2DM have insulin resistance and are at increased risk of developing cardiovascular disease (CVD) [2]. Diabetic dyslipidemia contributes to the excess morbidity and mortality in T2DM [2] and postprandial triglyceridemia is a distinct component of diabetic dyslipidemia [3]. Postprandial triglyceridemia is an independent risk factor for CVD in individuals with and without T2DM [4–6].

Obesity is associated with insulin resistance, which in turn is linked to atherogenic dyslipidemia, which postprandial hyperlipidemia is a major component of [7]. Atherosclerotic cardiovascular disease is one important consequence of the obesity pandemic that currently affects more than a billion people worldwide [8].

Thus, therapeutic approaches aiming to reduce postprandial lipid concentrations may reduce the cardiovascular mortality in patients with T2DM [9, 10].

Glucagon-like peptide-1 (GLP-1) is a gut incretin hormone secreted in response to nutrient ingestion [11]. It has several physiological effects mediated by the widely expressed GLP-1 receptor. Following binding to and activation of the GLP-1 receptor in pancreatic *β* cells, insulin secretion is elicited in a glucose dependent manner.

GLP-1 also delays gastric emptying and induces satiety, thus decreasing energy intake, which in turn leads to weight loss [12].

Therapeutic approaches based on the actions of the incretin hormone glucagon-like peptide GLP-1 have been widely established in the management of type 2 diabetes [13–15].

Interestingly, much less research has been aimed at elucidating the role of GLP-1 in lipid metabolism and in particular postprandial dyslipidemia, although preclinical models have provided some clues in this regard. Acute intravenous administration of the incretin hormone GLP-1 has been shown to lower postprandial triglyceride levels in healthy volunteers [16]. There are limited human data investigating the chronic effects of GLP-1 receptor agonists on postprandial triglyceride and lipoprotein concentrations.

Currently, two GLP-1 receptor agonists are available as subcutaneously administered treatment for T2DM, exenatide and liraglutide.

Long-term clinical trials with liraglutide or exenatide versus placebo have shown their ability to improve glycaemic control and reduce body weight [17], but their chronic effects on postprandial lipaemia have not been well investigated.

## 2. Materials and Methods

This was a single-center, randomized, interventional study in 20 patients with T2DM. All study subjects gave their informed consent. The study was conducted in the Diabetic Centre of the “Konstantopouleio” General Hospital, Nea Ionia, Athens, Greece, between April 2013 and November 2013. The study included obese men and women aged 18–80 years with T2DM who were treated with diet and exercise with or without a stable dose of metformin for at least 30 days prior to the start of the protocol. Patients were deemed obese if their body mass index (BMI) equaled or exceeded 30 kg/m^2^.

Main exclusion criteria included impaired renal function (creatinine clearance < 1.0 mL/s), known clinically significant active CVD, alcoholism/drug abuse, treatment with corticosteroids within 2 months, treatment with an investigational drug within 30 days, or current treatment with drugs known to affect gastrointestinal motility. None of the participants had any abnormalities, in terms of either (1) anaemia (haemoglobin < 120 g/L) or (2) an elevation of liver enzymes (alanine aminotransferase, aspartate aminotransferase, alkaline phosphatase, and *γ*-glutamyltransferase) to levels higher than double the respective normal value. Women who were pregnant or intended to become pregnant during the study and women who were breastfeeding were also excluded. Patients were excluded if they had clinically important medical conditions or had used thiazolidinediones, sulfonylureas, meglitinides, a-glucosidase inhibitors, pramlintide, exogenous insulin, lipid-lowering drugs, or weight-loss drugs within the prior 2 months. None of the participants had ever used DPP-4 inhibitors or GLP-1 analogues, nor did they have a history of gastrointestinal disorders or had previously undergone abdominal surgery. All the participants were nonsmokers.

Patients underwent a standardized meal tolerance test at baseline (visit 1—beginning of treatment) and after a two-week treatment period (visit 2). Meals provided for each patient were identical at the baseline and week-2 assessments. The standardized meal LIPOTEST (D.GENOMERES Medical Research, Athens, Greece) [18] was given in the form of 115 g powder diluted in water (150 mL water, final volume as mousse 265 mL). The meal consists of 75 g of fat, 25 g of carbohydrates, and 10 g of protein.

After at least a 10-h overnight fast, exenatide or liraglutide was administered 30 minutes prior to the standard meal, as described in previous studies. All participants were advised to maintain their usual dietary habits and to avoid strenuous exercise before the experiments. Each patient received a daily injection with either liraglutide or bidaily injection of exenatide according to the group each patient was randomized to. Individuals randomized to liraglutide underwent a weekly dose escalation, starting at 0.6 mg/day of subcutaneous (sc) injection for the first week followed by 1.2 mg/day for the second week. Exenatide treatment consisted of a 5 mg sc injection twice daily (bid) for the first week followed by 10 mg sc bid for the second week.

The patients were monitored at baseline as well as two weeks after the beginning of treatment. At each of the two visits the following parameters were recorded for each patient: serum cholesterol, high density lipoproteins (HDL), low density lipoproteins (LDL), triglycerides, insulin, and glucose. Each parameter was measured at 0 (fasting), 120, and 240 minutes after the ingestion of the lipid meal.

Furthermore the patient's body mass index (BMI), waist circumference, thigh circumference, and HbA1c were recorded.

For the statistical analysis we used the Mann-Whitney independent samples* U* test and Fisher's exact test to test for differences in means of the baseline characteristics between patients in the exenatide and liraglutide groups. ANOVA and ANCOVA for repeated measurements were performed to test the timing effect of the studied parameters after the test meal and the differences between exenatide and liraglutide at visit 1 and at visit 2, respectively. The Greenhouse-Geisser adjustment was used when the sphericity assumptions were not fulfilled. Postprandial responses over the 240-minute period were calculated as the area under the curve (AUC) using the trapezoid rule.

## 3. Results

The baseline characteristics of the patients in the two study groups are illustrated in Tables 1 and 2.

There was no statistically significant difference in age, HbA1c, waist-to-hip ratio, and BMI between the exenatide and liraglutide groups.

The fasting values for different parameters of the patients in the exenatide group and the liraglutide group for visits 1 and 2 are shown in Tables 3 and 4.

There was an increase in the postprandial total cholesterol levels in patients treated with both exenatide and liraglutide at visit 1, which was statistically significant. Postprandial triglycerides also showed an increase, but that did not reach statistical significance. HDL levels remained stable in the two measurements after the standard meal. Glucose in both groups and LDL in the liraglutide-treated patients showed a decrease in their levels, the former being statistically significant, the latter not. In contrast LDL in the exenatide group showed a marginal increase that was statistically significant. Postprandial insulin reached a peak value at 120 minutes after the mean to return to lower level at 240 minutes in both groups. There was no difference between any of the parameters, when comparing the exenatide and liraglutide groups.

At visit 2 triglycerides, HDL, and LDL increased at the two postprandial measurements as did the total cholesterol in the liraglutide group. To the contrary, total cholesterol in the exenatide group showed a decrease that was not statistically significant. Glucose levels showed a similar decrease in the first visit and insulin levels peaked at 120 minutes to level off at 240 minutes after the lipid meal. There was no difference for any of the parameters between the exenatide and liraglutide groups.

The AUC values for each parameter of the patients in the exenatide and liraglutide groups at visits 1 and 2 are shown in Table 5. There was no statistically significant difference for any of those between exenatide and liraglutide. The results remained the same even after correcting for possible confounding factors (age, WHR, HbA1c, and BMI).

## 4. Discussion

Acute intravenous administration of the incretin hormone GLP-1 has been shown to lower postprandial triglyceride levels in healthy volunteers [16]. The precise mechanism is yet to be determined, as many factors can influence circulating triglyceride concentrations [5, 19].

Short-term treatment with vildagliptin and sitagliptin, selective DPP-4 inhibitors, has been shown to reduce postprandial lipaemia in T2DM [20, 21], presumably by inhibiting the inactivation of endogenous GLP-1 by DPP-4.

Also, treatment with the GLP-1 receptor agonist exenatide reduces postprandial triglyceride and lipoprotein concentrations in T2DM, in the short term [22]. Previous studies proved that even a single dose of exenatide significantly lowers postprandial triglycerides levels [23]. The effect of exenatide on postprandial lipaemia may be influenced by several mechanisms mediated by GLP-1 receptor signaling, as well as by delayed gastric emptying [24].

Recently, Hermansen et al. investigated the effects of steady-state liraglutide 1.8 mg versus placebo on postprandial plasma lipid concentrations after 3 weeks of treatment in patients with T2DM. The conclusion of this study was that liraglutide treatment in patients with T2DM significantly reduced postprandial excursions of triglyceride and apolipoprotein B48 after a fat-rich meal. Reductions in postprandial glucose and glucagon responses, as well as in LDL and total cholesterol concentrations from baseline, were also observed with liraglutide. The effects of liraglutide on postprandial lipaemia appeared to be independent of gastric emptying [25].

The present study is the first to compare the effects of exenatide and liraglutide on postprandial lipaemia both in short and in long term in obese patients with T2DM.

Although exenatide and liraglutide share the same basic mechanisms of action, differences in pharmacokinetic and pharmacodynamic characteristics translate into differential effects on parameters of fasting and postprandial glycaemia [26] and presumably of postprandial lipaemia.

A deceleration of gastric emptying by GLP-1 has been demonstrated in patients with type 2 diabetes and healthy individuals [27–29]. It has also been reported that the GLP-1 induced deceleration of gastric emptying is significantly diminished already after 5 h of continuous infusion compared with its initial effects. It has also been shown that postprandial glucose control by GLP-1 is attenuated by its chronic administration [30].

GLP-1 seems to inhibit gastric emptying primarily through inhibition of the vagal nerve [31], but this mechanism is subject to rapid tachyphylaxis after chronic GLP-1 exposure [30].

The difference in the duration of action between bidaily exenatide and liraglutide influences gastric emptying. Therefore, delayed gastric emptying might be attenuated with long-acting GLP-1 analogs such as liraglutide compared to short-acting GLP-1 analogs such as exenatide [32]. Indeed, gastric emptying time was more prolonged with exenatide compared to long-acting release exenatide (LAR), which is administered once weekly, in patients with type 2 diabetes [33]. In addition to the different durations of action, different immunogenicity profiles across different classes of GLP-1 analogs could affect drug efficacy and safety profiles [34].

In a recent study, the acute and chronic effects of exenatide twice daily and liraglutide on gastric emptying were examined in rats. Gastric emptying was assessed using a standard acetaminophen release assay. After the acute test, rats were administered either exenatide bidaily or daily liraglutide for 14 days. On day 14, the gastric emptying rate was reassessed. While both compounds exerted robust acute reductions in gastric emptying, the effect was markedly diminished following 14 days of liraglutide administration. In contrast, exenatide-treated rats still displayed a profound reduction in gastric emptying at the 14-day time-point [35]. The data suggest that the “gastric inhibitory” GLP-1 receptors in rats are subject to desensitization-tachyphylaxis but this effect seems to be dependent on full 24-h exposure as obtained by liraglutide.

Other studies showed equivalence in gastric emptying between liraglutide and placebo over the full postprandial period after short-term treatment [36], but slower gastric emptying with liraglutide during the initial hour [37].

In our study we demonstrated that there is no difference between the two treatments on postprandial lipaemia both on first administration and after a two-week treatment. These findings suggest that there are mechanisms beyond the inhibition of gastric emptying that participate in the regulation of lipid postprandial metabolism following the treatment with liraglutide or exenatide.

We are well aware of the limitations of our study, which we consider to be the small number of participants and its short duration. However, we consider that the similarity of the two treatment groups in terms of baseline characteristics is an important advantage.

In conclusion, exenatide and liraglutide both appear to be equally effective in lowering postprandial lipaemia after the first administration and after a two-week treatment. Further investigation might be needed to elucidate the mechanisms which lead to this phenomenon, which seem to be independent of gastric emptying.

## Figures and Tables

**Table 1:** Demographic and clinical characteristics of the study subjects stratified according to treatment.

Characteristics	Exenatide (*n* = 10)	Liraglutide (*n* = 10)	*P*
Age (years)	45.2 ± 11.4	54.6 ± 12.9	0.143
Diabetes duration (years)	2.40 ± 2.17	2.50 ± 2.32	0.922
Hypertension	3 (yes)/7 (no)	4 (yes)/6 (no)	1.000∗
Metformin use	9 (yes)/1 (no)	10 (yes)/0 (no)	1.000∗
HbA1c (mmol/mol)	41.53 ± 10.17	42.74 ± 6.97	0.912
WHR (waist-to-hip ratio)	1.01 ± 0.13	0.98 ± 0.08	0.393
BMI (kg/m^2^)	37.18 ± 5.82	37.11 ± 5.09	1.000
HbA1c (mmol/mol)	41.53 ± 10.17	42.73 ± 6.97	0.915

Data are means ± SD. (*P* indicates the result of the Mann-Whitney test between the two groups; *the result of Fisher's exact test for differences between the two groups).

**Table 2:** Baseline measurements of the study subjects stratified according to treatment.

Characteristics	Exenatide (*n* = 10)	Liraglutide (*n* = 10)	*P*
Glucose (mg/dL)	7.34 ± 2.73	6.69 ± 0.94	0.971
Total cholesterol (mg/dL)	5.05 ± 1.42	5.15 ± 1.19	0.853
Triglycerides (mg/dL)	3.03 ± 4.69	2.42 ± 2.97	0.853
HDL (mg/dL)	1.03 ± 0.33	1.19 ± 0.45	0.481
LDL (mg/dL)	3.00 ± 1.26	2.87 ± 0.71	1.000
Insulin (mU/L)	159.86 ± 84.58	152.07 ± 49.74	0.739

Data are means ± SD. (*P* indicates the result of the Mann-Whitney test between the two groups).

**Table 3:** Fasting and postprandial profiles of the measured parameters in patients receiving exenatide and liraglutide on visit 1.

Parameters	0	120	240	*P*	P*
Triglycerides (mmol/L)					
Exenatide	3.03 ± 4.69	3.18 ± 4.24	3.48 ± 4.23	0.185	0.147
Liraglutide	2.42 ± 2.97	2.47 ± 2.47	2.53 ± 2.59	0.628	
Total cholesterol (mmol/L)					
Exenatide	5.05 ± 1.42	5.06 ± 1.41	5.20 ± 1.50	0.048	0.232
Liraglutide	5.15 ± 1.19	5.00 ± 1.00	5.24 ± 1.16	0.016	
HDL (mmol/L)					
Exenatide	1.03 ± 0.33	1.04 ± 0.30	1.03 ± 0.33	0.622	0.392
Liraglutide	1.19 ± 0.45	1.17 ± 0.44	1.19 ± 0.45	0.597	
LDL (mmol/L)					
Exenatide	3.00 ± 1.26	3.00 ± 1.23	3.04 ± 1.29	0.041	0.461
Liraglutide	2.87 ± 0.71	2.80 ± 0.63	2.85 ± 0.63	0.187	
Glucose (mmol/L)					
Exenatide	7.33 ± 2.73	6.50 ± 1.44	5.91 ± 1.84	0.032	0.803
Liraglutide	6.69 ± 0.93	6.06 ± 1.20	5.08 ± 0.53	<0.0001	
Insulin (pmol/L)					
Exenatide	159.86 ± 84.58	232.67 ± 131.82	153.00 ± 117.10	0.006	0.424
Liraglutide	152.07 ± 49.74	208.39 ± 111.16	105.07 ± 63.80	0.002	

Data are means ± SD. *P* indicates the result of ANOVA for repeated measurements within each group (*P* value for the effect of time); P* indicates the result of ANOVA for repeated measurements between the two groups (exenatide versus liraglutide) (time × group interaction).

**Table 4:** Fasting and postprandial profiles of the measured parameters in patients receiving exenatide and liraglutide on visit 2.

Parameters	0	120	240	*P*	P*
Triglycerides (mmol/L)					
Exenatide	2.16 ± 2.42	2.42 ± 2.37	2.50 ± 2.13	0.040	0.523
Liraglutide	1.72 ± 1.00	1.92 ± 0.94	1.89 ± 0.88	0.030	
Total cholesterol (mmol/L)					
Exenatide	4.73 ± 1.22	4.72 ± 1.22	4.38 ± 1.87	0.594	0.464
Liraglutide	4.69 ± 1.13	4.70 ± 0.01	4.82 ± 1.03	0.161	
HDL (mmol/L)					
Exenatide	1.00 ± 0.28	1.01 ± 0.28	1.05 ± 0.28	0.017	0.493
Liraglutide	1.19 ± 0.41	1.17 ± 0.42	1.20 ± 0.43	0.572	
LDL (mmol/L)					
Exenatide	2.91 ± 1.13	2.92 ± 1.17	3.10 ± 1.29	0.002	0.105
Liraglutide	2.75 ± 0.97	2.71 ± 0.92	2.81 ± 0.90	0.150	
Glucose (mmol/L)					
Exenatide	6.42 ± 1.46	6.80 ± 2.36	6.07 ± 1.88	0.069	0.710
Liraglutide	6.02 ± 1.15	6.15 ± 1.50	5.30 ± 0.61	0.066	
Insulin (pmol/L)					
Exenatide	150.75 ± 87.46	233.15 ± 182.40	172.93 ± 175.63	0.143	0.081
Liraglutide	144.53 ± 72.94	413.56 ± 350.00	127.91 ± 84.20	0.026	

Data are means ± SD. *P* indicates the result of ANOVA for repeated measurements within each group (*P* value for the effect of time); P* indicates the result of ANOVA for repeated measurements between the two groups (exenatide versus liraglutide) (time × group interaction).

**Table 5:** Total AUC values in patients receiving exenatide and liraglutide on visits 1 and 2.

Parameters	Exenatide	Liraglutide	*P*
First visit			
Triglycerides (mmol/L*·*min)	771.50 ± 1041.54	593.39 ± 628.76	0.290
Total cholesterol (mmol/L*·*min)	1221.13 ± 343.35	1223.00 ± 258.93	0.140
HDL (mmol/L*·*min)	248.80 ± 74.93	284.07 ± 106.98	0.306
LDL (mmol/L*·*min)	716.39 ± 300.32	679.56 ± 155.12	0.472
Glucose (mmol/L*·*min)	1575.42 ± 408.90	1433.90 ± 206.56	0.364
Insulin (pmol/L*·*min)	46692.36 ± 26383.91	40435.20 ± 19466.77	0.151
Second visit			
Triglycerides (mmol/L*·*min)	569.18 ± 556.38	446.46 ± 224.27	0.307
Total cholesterol (mmol/L*·*min)	1112.75 ± 266.78	1134.73 ± 249.96	0.850
HDL (mmol/L*·*min)	244.76 ± 67.94	283.61 ± 100.85	0.305
LDL (mmol/L*·*min)	711.89 ± 280.85	660.29 ± 221.26	0.089
Glucose (mmol/L*·*min)	1565.43 ± 477.52	1416.92 ± 252.87	0.762
Insulin (pmol/L*·*min)	47399.76 ± 35383.95	65973.24 ± 45256.94	0.174

Data are means ± SD. *P* indicates the result of the Mann-Whitney *U* test.
